# Cerebral blood flow, amyloid burden, and cognition in cognitively normal individuals

**DOI:** 10.1007/s00259-022-05958-8

**Published:** 2022-09-08

**Authors:** Jarith L. Ebenau, Denise Visser, Sander C. J. Verfaillie, Tessa Timmers, Mardou S. S. A. van Leeuwenstijn, Mara ten Kate, Albert D. Windhorst, Frederik Barkhof, Philip Scheltens, Niels D. Prins, Ronald Boellaard, Wiesje M. van der Flier, Bart N. M. van Berckel

**Affiliations:** 1 Alzheimer Centre, Department of Neurology, Amsterdam Neuroscience, Vrije Universiteit Amsterdam, Amsterdam UMC, De Boelelaan 1118, 1081 HZ Amsterdam, The Netherlands; 2 Department of Radiology & Nuclear Medicine, Amsterdam Neuroscience, Vrije Universiteit Amsterdam, Amsterdam UMC, Amsterdam, The Netherlands; 3 UCL Institutes of Neurology and Healthcare Engineering, London, UK; 4 Brain Research Centre, Amsterdam, The Netherlands; 5 Department of Epidemiology & Data Science, Vrije Universiteit Amsterdam, Amsterdam UMC, Amsterdam, The Netherlands

**Keywords:** Alzheimer’s disease, Cerebral blood flow, Amyloid, PET, *R*_1_

## Abstract

**Purpose:**

The role of cerebral blood flow (CBF) in the early stages of Alzheimer’s disease is complex and largely unknown. We investigated cross-sectional and longitudinal associations between CBF, amyloid burden, and cognition, in cognitively normal individuals with subjective cognitive decline (SCD).

**Methods:**

We included 187 cognitively normal individuals with SCD from the SCIENCe project (65 ± 8 years, 39% F, MMSE 29 ± 1). Each underwent a dynamic (0–70 min) [^18^F]florbetapir PET and T1-weighted MRI scan, enabling calculation of mean binding potential (BP_ND_; specific amyloid binding) and *R*_1_ (measure of relative (r)CBF). Eighty-three individuals underwent a second [^18^F]florbetapir PET (2.6 ± 0.7 years). Participants annually underwent neuropsychological assessment (follow-up time 3.8 ± 3.1 years; number of observations *n* = 774).

**Results:**

A low baseline *R*_1_ was associated with steeper decline on tests addressing memory, attention, and global cognition (range betas 0.01 to 0.27, *p* < 0.05). High BP_ND_ was associated with steeper decline on tests covering all domains (range betas − 0.004 to − 0.70, *p* < 0.05). When both predictors were simultaneously added to the model, associations remained essentially unchanged. Additionally, we found longitudinal associations between *R*_1_ and BP_ND_. High baseline BP_ND_ predicted decline over time in *R*_1_ (all regions, range betas_BP×time_ − 0.09 to − 0.14, *p* < 0.05). Vice versa, low baseline *R*_1_ predicted increase in BP_ND_ in frontal, temporal, and composite ROIs over time (range betas_*R*1×time_ − 0.03 to − 0.08, *p* < 0.05).

**Conclusion:**

Our results suggest that amyloid accumulation and decrease in rCBF are two parallel disease processes without a fixed order, both providing unique predictive information for cognitive decline and each process enhancing the other longitudinally.

## Introduction

Deposition of amyloid β (Aβ) into plaques is one of the main pathophysiological hallmarks of Alzheimer’s disease (AD) and an important determinant of cognitive decline [1, 2].

Amyloid accumulation is thought to be the primary event in the pathogenesis of AD, initiating a series of events that ultimately result in neuronal damage and dementia [3, 4]. The process of amyloid accumulation starts decades before the onset of dementia, and amyloid plaques can already be present in cognitively normal individuals [5]. Although amyloid pathology is an important marker of AD, other pathological processes also play a role, such as tau pathology, neuroinflammation, and changes in cerebral blood flow (CBF). Indeed, CBF is shown to be abnormal in AD dementia and relates to changes in brain glucose metabolism and synaptic failure [6–8]. Several studies using measures of CBF such as arterial spin labelling (ASL) MRI, early phase amyloid PET, or [^15^O]H_2_O PET found a lower CBF to be associated with advancing disease stage [9–14]. It is currently unclear how CBF and amyloid burden are interrelated, especially in the early stages of the disease, as previous studies provide conflicting results and are hampered by small sample sizes [12, 15–21]. In addition, most studies used a cross-sectional design, precluding the investigation of longitudinal trajectories. Only one study investigated CBF longitudinally and found both increases and decreases in CBF in amyloid-positive individuals [16]. CBF has furthermore been shown to be associated with cognition, but studies provide conflicting results and longitudinal studies are scarce [22–24].

Amyloid burden and CBF can be assessed simultaneously in vivo with dynamic [^18^F]florbetapir PET scans. Dynamic scanning provides two unique parameters of interest: (i) binding potential (BP_ND_), which is an exact quantification of specific binding to Aβ [25], and (ii) *R*_1_, which represents the ratio between *K*_1_ (the rate constant for ligand transfer from plasma to tissue) in the target region and the reference region, and which can be seen as a measure for relative CBF (rCBF) [10, 26, 27]. As a result, one [^18^F]florbetapir PET scan provides quantitative information on both amyloid load and rCBF.

This study focused on cognitively normal individuals who experienced cognitive complaints. These individuals presented to a memory clinic which makes them a clinically relevant population to study early amyloid pathology and the role of rCBF in the development of AD pathology. We hypothesized that both high amyloid burden and low rCBF are related to cognitive decline. Since CBF seems to decline in advancing disease stages, we hypothesized that a higher baseline amyloid burden is related to a subsequent decline in rCBF. The aims of this study were (1) to assess the association between amyloid burden, rCBF, and cognitive decline over time and (2) to investigate the relationship between (rate of accumulation of) amyloid burden and (rate of change in) rCBF in a relatively large sample of cognitively normal individuals.

## Material and methods

### Population

We included 187 cognitively normal individuals from the SCIENCe cohort, which is part of the Amsterdam Dementia Cohort [28, 29]. All participants with available [^18^F]florbetapir PET and MRI and available cognitive data were included. PET scans used in this study were acquired between 2015 and 2021. One hundred and seventy-five individuals were referred to the memory clinic by their general physician, a geriatrician, or a neurologist. All underwent an extensive standardized diagnostic work-up, including a neurological and neuropsychological examination, laboratory testing, and brain MRI, which was read by an experienced neuroradiologist. In a consensus meeting, individuals were categorized as having subjective cognitive decline when clinical and cognitive investigations were normal and criteria were not met for mild cognitive impairment (MCI) or dementia, nor for other neurological or psychiatric diseases that could be the cause of cognitive complaints. Participants were followed up annually, during which neuropsychological testing and clinical investigation were repeated. In addition, 12 participants were included via the Dutch Brain Research Registry (hersenonderzoek.nl). All experienced cognitive complaints in the absence of objective impairment and all received the same baseline work-up.

### Image acquisition

PET scans were acquired on an Ingenuity TF PET-CT (*n* = 140) or a Gemini TF PET-CT (*n* = 47; Philips, Best, the Netherlands) scanner which were calibrated to each other. Dynamic PET scans of 90 min (*n* = 140) were obtained starting simultaneously with tracer injection of approximately 370 MBq [^18^F]florbetapir. During the course of the study, we demonstrated that scan duration could be reduced without compromising the reliability of results [25]. Therefore, subsequent scans had a duration of 70 min (*n* = 43). The scan was terminated early in four instances due to participant-related issues (three after 60 min, one after 79 min). These scans were nonetheless included because quantification was reliable in these subjects with relatively low amyloid load [25].

Follow-up scans were available for *n* = 83 (44%) (*n* = 17 90-min scan; *n* = 66 70-min scan). Mean time between the two PET scans was 2.6 ± 0.7 years.

In addition, all individuals underwent structural MRI. The protocol included 3D T1-weighted images, 3D T2-weighted images, and 3D T2-weighted fluid-attenuated inversion-recovery (FLAIR) images [30].

### Image analysis

Data were reconstructed while using standard LOR RAMLA reconstruction algorithm with corrections for scatter, random coincidences, attenuation, decay, and dead time. Images were reconstructed with a matrix size of 128 × 128 × 90 and a voxel size of 2 × 2 × 2 mm^3^. Isotropic 3-dimensional T1-weighted MR images were co-registered to PET images using Vinci software (Max Planck Institute, Cologne, Germany). Next, regions of interest (ROIs) were defined on the co-registered MRI using the probabilistic Hammers brain atlas in PVElab [31]. Receptor parametric mapping (RPM) was used to generate parametric BP_ND_ and *R*_1_ images with cerebellar gray matter as a reference region using PPET [25, 32–34]. We calculated (volume weighted) mean cortical BP_ND_ and *R*_1_ in the following (bilateral) ROIs: frontal, temporal, parietal, occipital, and a composite ROI consisting of orbitofrontal, temporal, parietal, anterior cingulate, posterior cingulate, and precuneus regions [35]. The difference in time between MRI and PET was generally within 1 year (median time difference 0.2 years (IQR − 0.5 – 0.5)).

White matter hyperintensities were visually assessed using the Fazekas scale (range 0–3) [36]. Microbleeds were assessed on T2-weighted images and defined as small dot-like hypointense lesions. They were counted and dichotomized into absent (0) or present (≥ 1 microbleed). Scans were reviewed by a neuroradiologist.

### Neuropsychological assessment

All participants underwent extensive standardized neuropsychological assessments [30]. For memory, we used the Visual Association Test version A (VAT-A) and the total immediate and delayed recall condition of the Dutch version of the Rey Auditory Verbal Learning Task (RAVLT). To assess language, we used category fluency (animals). For attention, we used the Trail Making Test A (TMT-A) and Stroop tasks I and II (naming and color naming). For executive functioning, we used the TMT-B and Stroop task III (color-word). For global cognition, we used the Mini-Mental State Examination (MMSE). Raw test scores for TMT and Stroop were log transformed, because data were right-skewed. Values were subsequently inverted, so that a lower score implies worse test performance for all tests. We used available test results of visits before as well as after the PET scan, in order to accurately estimate the cognitive slope [37]. This resulted in longitudinal cognitive data covering 3.8 ± 3.1 years. Concurrent time points were defined as the visit closest to the date of the baseline PET scan (median − 0.19, IQR − 0.41 to 0.00). In total, 774 neuropsychological investigations of 187 patients were available (165 ≥ 2 visits, median 3).

### Statistics

All analyses were performed in R version 4.0.3. For all analyses, BP_ND_ and *R*_1_ were transformed into *Z*-scores, for comparability of effect sizes. *Z*-scores were based on baseline PET scans (*n* = 187). We used the false discovery rate (FDR) to correct for multiple testing, and FDR corrected *p* values < 0.05 were considered significant. We used linear mixed models (LMM) with time as determinant to estimate slopes for imaging measures and cognitive tests for the whole group.

First, we investigated the relationship between baseline *R*_1_, baseline BP_ND_, and cognitive test performance, using LMM. For this set of models, the composite ROI was used. Model 1 included *R*_1_, time, and *R*_1_ × time as predictors and cognitive test results as outcome. Separate models were run with different cognitive tests as outcome measure (*n* = 10 neuropsychological tests). Next, we repeated the analyses with BP_ND_ instead of *R*_1_ as predictor (model 2). Then we included both *R*_1_ and BP_ND_ as predictors in the model (predictors: *R*_1_, BP_ND_, time, *R*_*1*_ × time, BP_ND_ × time; model 3). When we ran model 3, we tested whether there was an interaction between *R*_1_ × BP_ND_ × time for all neuropsychological tests. When this interaction term was significant, we provide the betas full model including the three-way interaction term. When the three-way interaction was not significant, it was removed from the model. All models were corrected for age, sex, education, and PET and MRI scanner type. Models included a random intercept, and a random slope if this improved the model fit, which was the case for RAVLT immediate, RAVLT delayed, Stroop II, Stroop III, and MMSE.

We then used LMM to assess the cross-sectional and longitudinal relationship between BP_ND_ and *R*_1_. We first assessed the effect of baseline BP_ND_ on *R*_1_. Model 1 included baseline BP_ND_, time, and BP_ND_ × time as predictors, and *R*_1_ as outcome (including baseline and follow-up *R*_1_ values). Model 2 was additionally corrected for age, sex, and PET and MRI scanner type. In the models, “BP_ND_” represents the effect of BP_ND_ on *R*_1_, when time = 0. The interaction term “BP_ND_ × time” reflects the effect of BP_ND_ on annual change in *R*_1_. We analyzed the associations in frontal, temporal, parietal, occipital, and composite regions separately, such that for each analysis, the ROI used for BP_ND_ was the same as the ROI used for *R*_1_. Subsequently, we performed an additional set of analyses, where predictors and outcome were reversed so that baseline *R*_1_, time, and *R*_1_ × time were used as predictors, and longitudinal BP_ND_ as outcome. Models included a random intercept.

For visualization in the figures, we used tertiles to divide our sample based, on *R*_1_ (low, intermediate, and high baseline *R*_1_) and BP_ND_ (low, intermediate, and high baseline BP_ND_).

## Results

### Demographics

Table 1 shows the baseline demographics of the sample. One hundred and eighty-seven individuals were on average 64.6 ± 7.6 years old, 74 (39%) were female, MMSE was 28.9 ± 1.2, and 69 (39%) were APOE4 carrier. Fazekas score and number of microbleeds were low. During follow-up, 13 individuals progressed to MCI or dementia (MCI *n* = 9, AD dementia *n* = 2, dementia with Lewy bodies *n* = 1, vascular dementia *n* = 1).

**Table 1:** Baseline demographics

		Total *N* = 187	
Demographics	Age, mean ± SD	64.61 ± 7.61	
	Sex, *n* female (%)	73 (39.04%)	
	Education, median [IQR]	6 [5–6]	
	APOE4 carriership, *n* (%)	69 (39.20%)	
	Amyloid positivity, *n* (%)†	45 (24.06%)	
	Fazekas, mean ± SD	0.88 ± 0.79	
	Microbleeds, *n* (%)‡	32 (17.11%)	
	Clinical progression, *n* (%)¤	13 (6.95%)	
		Concurrent	Annual change
Amyloid burden	BP_ND_ frontal	0.18 (0.012)	0.02 (0.003)*
	BP_ND_ temporal	0.13 (0.009)	0.01 (0.002)*
	BP_ND_ parietal	0.21 (0.012)	0.01 (0.002)*
	BP_ND_ occipital	0.20 (0.009)	0.00 (0.002)
	BP_ND_ composite	0.17 (0.010)	0.01 (0.002)*
Relative cerebral blood flow	*R*_1_ frontal	0.93 (0.004)	0.01 (0.002)*
	*R*_1_ temporal	0.89 (0.004)	0.003 (0.001)*
	*R*_1_ parietal	0.95 (0.004)	− 0.003 (0.002)*
	*R*_1_ occipital	0.98 (0.004)	− 0.003 (0.002)
	*R*_1_ composite	0.92 (0.004)	0.002 (0.010)
Neuropsychological tests	VAT-A	11.53 (0.053)	− 0.04 (0.013)*
	RAVLT immediate	44.77 (0.594)	0.62 (0.150)*
	RAVLT delayed	8.99 (0.196)	0.11 (0.048)*
	Animal fluency	23.56 (0.364)	0.02 (0.061)
	TMT-A§	− 3.47 (0.020)	− 0.001 (0.003)
	TMT-B§	− 4.32 (0.024)	− 0.004 (0.003)
	Stroop I§	− 3.75 (0.011)	0.002 (0.002)
	Stroop II§	− 4.05 (0.012)	0.01 (0.002)*
	Stroop III§	− 4.52 (0.016)	0.02 (0.003)*
	MMSE	28.76 (0.071)	0.05 (0.024)

Baseline demographics in the total sample. Data is presented as mean (SE) unless otherwise specified. Values for BP_ND_, *R*_1_, and cognitive test results do not represent the observed data but are obtained using linear mixed models with time as only predictor (intercept as concurrent value at time of baseline PET scan; beta associated with time as value for annual change). Annual change for BP_ND_ and *R*_1_ is based on the subset with an available follow-up PET (*n* = 83)

^*^p value < 0.05

^†^Amyloid positivity as determined by visual assessment of baseline [^18^F]florbetapir PET

^‡^Values are dichotomized into 0 counts and ≥ 1 counts; *n* shown is number of participants with ≥ 1 count

^§^Values are log transformed and inverted such that a lower score implies a worse test result, complicating interpretation of values

^¤^Clinical progression to mild cognitive impairment *n* = 9, *AD* dementia *n* = 2, dementia with Lewy bodies *n* = 1, vascular dementia *n* = 1

*BP*_*ND*_ binding potential, *VAT* Visual Association Test, *RAVLT* Rey Auditory Verbal Learning Task, *TMT* Trail Making Test, *MMSE* Mini-Mental State Examination

Eighty-three individuals underwent follow-up PET. Individuals in this subset were similar to the total sample in terms of baseline demographics. In this cognitively normal group, we found on average only very subtle changes over time. BP_ND_ increased over time in frontal, temporal, parietal, and composite regions, with the greatest change in the frontal region (beta 0.02 (SE 0.003)). On average, *R*_1_ also increased over time in frontal and temporal regions (0.01 (0.002); 0.003 (0.001)), but decreased subtly in the parietal region (− 0.003 (0.002)). Baseline neuropsychological test scores were within age- and education-dependent norms. Over time and for the whole sample, we found a small improvement on RAVLT immediate, RAVLT delayed, Stroop II, and Stroop III, while the score on VAT-A became somewhat lower over time.

### Relationship between BP_ND_, *R*_1_, and cognition

**Fig. 1:**
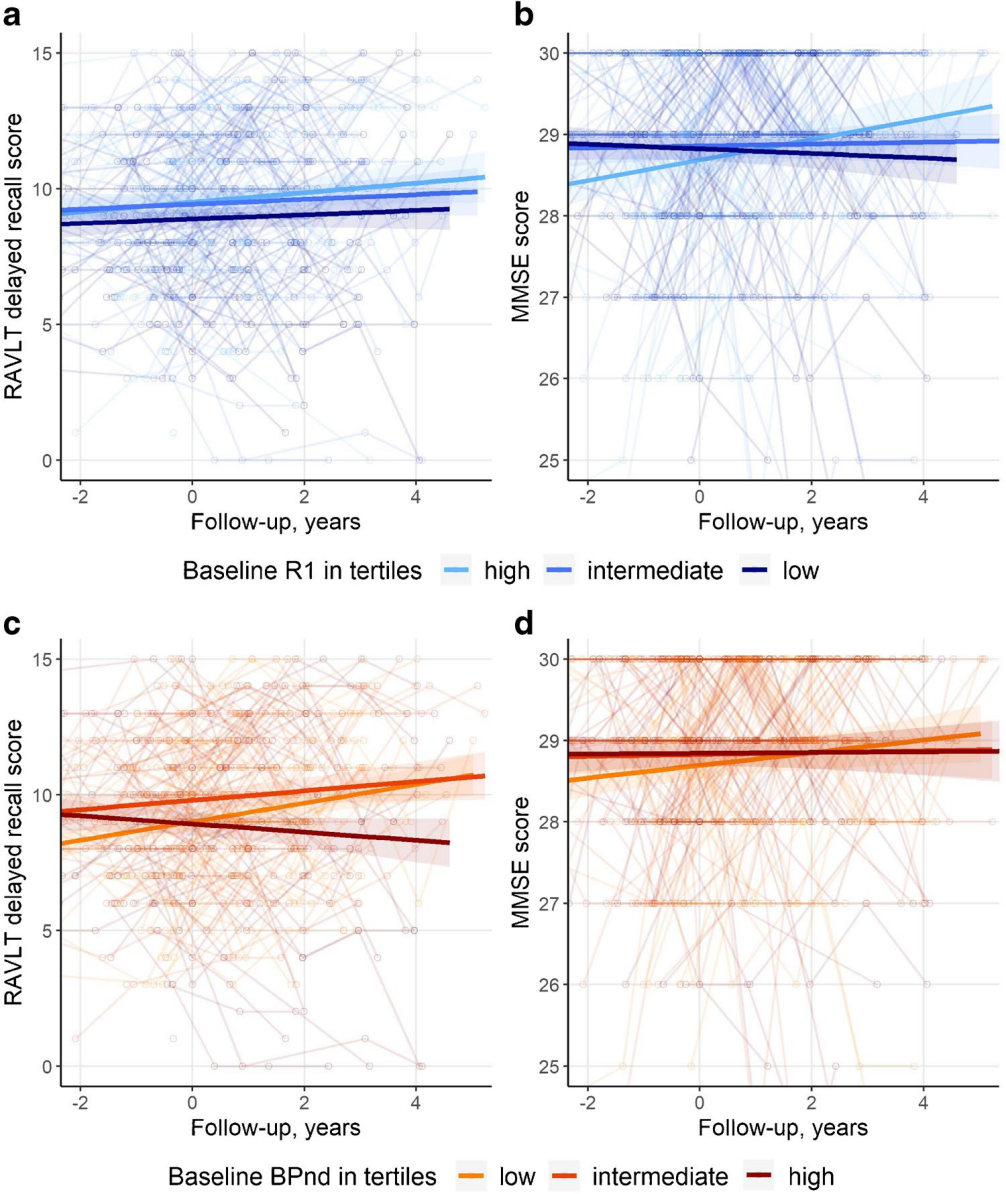
Neuropsychological test performance over time. Spaghetti plots showing individual neuropsychological trajectories on two neuropsychological tests. Rey Auditory Verbal Learning Task delayed recall (**a** and **c**) and mini mental state examination (**b** and **d**). For visualization, the sample was divided into tertiles. Separate lines represent the unadjusted mean trajectory for each *R*_1_ tertile separately (**a** and **b**, based on baseline *R*_1_ values) and for each BP_ND_ tertile separately (**c** and **d**, based on baseline BP_ND_ values). Figures represent raw test scores

We first analyzed how both baseline PET markers were associated with cognitive performance (Table 2, Fig. 1). We did not find any cross-sectional associations between *R*_1_ and any of the cognitive tests (model 1). By contrast, we found effects of *R*_1_ on slope of several tests (*p* for interaction with time < 0.05). A lower baseline *R*_1_ was associated with a worse trajectory for tests for memory (RAVLT immediate and delayed), attention (TMT-A), and global cognition (MMSE), but not for tests in other cognitive domains. The results did not remain significant after correction for multiple testing. Next, we used baseline BP_ND_ as predictor in our models (model 2). We only found a cross-sectional association between a higher baseline BP_ND_ and a lower MMSE score. By contrast, we found effects of BP_ND_ on slope for a large number of tests. After correction for multiple testing, associations between BP_ND_ and cognitive slope remained significant for RAVLT immediate, RAVLT delayed, Animal Fluency, and Stroops I–III. When we simultaneously entered BP_ND_ and *R*_1_ in model 3, results remained essentially unchanged. Testing for interactions between the two measures of Alzheimer pathology, we found an interaction between *R*_1_ and BP_ND_ for slope of RAVLT immediate (beta_*R*1_ × _BP_ × _time_ − 0.23 (SE 0.09)), such that the effect of *R*_1_ on RAVLT immediate slopes was mainly present in individuals with low BP_ND_. There was no interaction for any of the other neuropsychological tests, implying independent effects on cognitive decline of BP_ND_ and *R*_1_.

**Table 2:** Relationship between *R*_1_, BP_ND_, and cognition in composite region of interest

	Concurrent				Longitudinal			
	Model 1	Model 2	Model 3		Model 1	Model 2	Model 3	
	*R*_1_	BP_ND_	*R*_1_	BP_ND_	*R*_1_ × time	BP_ND_ × time	*R*_1_ × time	BP_ND_ × time
VAT-A	− 0.09 (0.05)	− 0.07 (0.05)	− 0.09 (0.05)	− 0.07 (0.05)	0.01 (0.01)	− 0.02 (0.01)	0.01 (0.01)	− 0.02 (0.01)
RAVLT immediate	0.04 (0.59)	− 0.77 (0.58)	0.05 (0.59)	− 0.79 (0.59)	**0.27 (0.13)**	− 0.70 (0.12)*	0.29 (0.11)*†	− 0.70 (0.12)*†
RAVLT delayed	0.01 (0.20)	− **0.41 (0.20)**	− 0.0001 (0.20)	− **0.40 (0.20)**	**0.09 (0.04)**	− 0.24 (0.04)*	**0.08 (0.03)**	− 0.23 (0.04)*
Animal fluency	0.01 (0.36)	− 0.41 (0.36)	0.04 (0.36)	− 0.41 (0.36)	0.04 (0.05)	− 0.22 (0.06)*	0.04 (0.05)	− 0.22 (0.06)*
TMT-A	0.01 (0.02)	− 0.01 (0.02)	0.01 (0.02)	− 0.01 (0.02)	**0.01 (0.003)**	− 0.01 (0.003)	**0.01 (0.003)**	− 0.01 (0.003)
TMT-B	0.001 (0.02)	− 0.03 (0.02)	0.002 (0.02)	− 0.03 (0.02)	0.003 (0.003)	− **0.01 (0.003)**	0.003 (0.003)	− **0.01 (0.003)**
Stroop I	0.0001 (0.01)	− 0.01 (0.01)	0.001 (0.01)	− 0.01 (0.01)	0.002 (0.001)	− 0.004 (0.002)*	0.002 (0.001)	− 0.004 (0.002)*
Stroop II	0.0004 (0.01)	− 0.01 (0.01)	0.0003 (0.01)	− 0.01 (0.01)	0.002 (0.002)	− 0.01 (0.002)*	0.002 (0.002)	− 0.01 (0.002)*
Stroop III	− 0.004 (0.01)	− 0.002 (0.02)	− 0.01 (0.01)	− 0.002 (0.02)	− 0.001 (0.003)	− 0.01 (0.003)*	− 0.002 (0.003)	− 0.01 (0.003)*
MMSE	− 0.08 (0.07)	− **0.21 (0.07)**	− 0.08 (0.07)	− **0.21 (0.07)**	**0.04 (0.02)**	− **0.05 (0.02)**	0.04 (0.02)	− **0.05 (0.02)**

Values given are beta (SE) obtained via linear mixed models. Outcome: cognitive test performance. Predictors: Model 1: *R*_1_, time, *R*_1_ × time; Model 2: BP_ND_, time, BP_ND_ × time; Model 3: *R*_1_, BP_ND_, time, *R*_1_ × time, BP_ND_ × time. For all neuropsychological tests as outcome, we tested whether the interaction term *R*_1_ × BP_ND_ × time was significant. When this was the case, we show the betas of *R*_1_ × time and BP_ND_ × time of the model that included this three-way interaction term. All models were corrected for age, sex, education, and PET and MRI scanner type. Betas reflect the association with annual decline (interaction between predictor and time). Models included a random intercept, and a random slope was added for RAVLT immediate, RAVLT delayed, Stroop II, Stroop III, and MMSE. TMT and Stroop were log transformed and inverted. *R*_1_ and BP_ND_ are z-transformed

Bold *p* value < 0.05; *FDR corrected *p* value < 0.05

^†^interaction term *R*_1_ × BP_ND_ × time *p* value < 0.05

*SE* standard error, *VAT* Visual Association Test, *RAVLT* Rey Auditory Verbal Learning Task, *TMT* Trail Making Test, *MMSE* Mini-Mental State Examination

### Relationship between BP_ND_ and *R*_1_

**Fig. 2:**
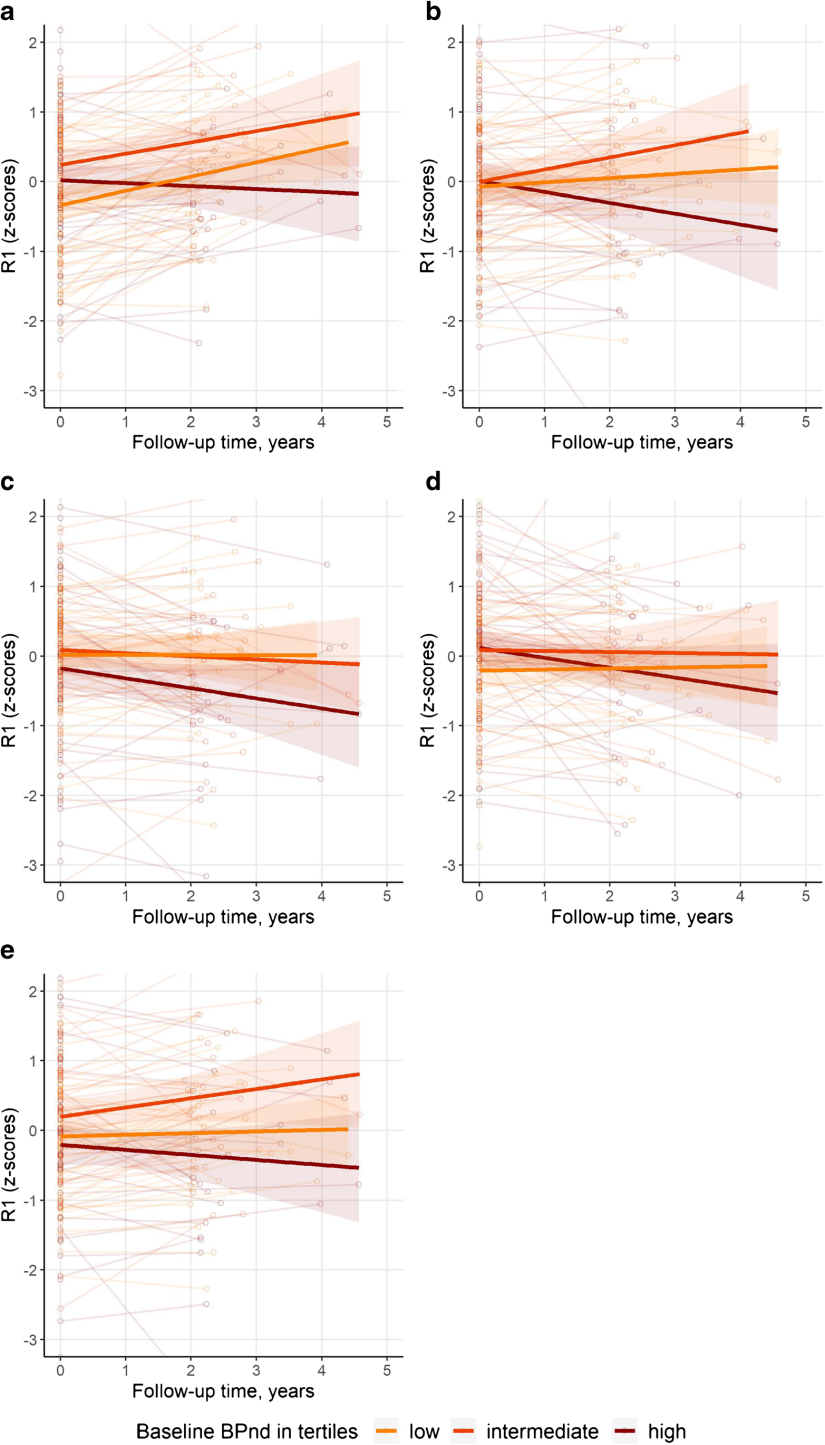
Visualization of trajectories of *R*_1_ over time. Spaghetti plots showing individual trajectories of *R*_1_ over time (**a** Frontal, **b** Temporal, **c** Parietal, **d** Occipital, **e** Composite regions). For visualization, the sample was divided into tertiles. Separate lines represent BP_ND_ tertiles (based on baseline values). BP_ND_ and *R*_1_ are z-transformed

Next, we investigated the relationship between BP_ND_ and *R*_1_. We first investigated the effect of BP_ND_ on *R*_1_ (Table 3). Somewhat counterintuitively, higher baseline values for occipital BP_ND_ were associated with higher baseline values for occipital *R*_1_ (Table 3). There were no cross-sectional associations in other regions. By contrast, longitudinally, in all regions higher baseline BP_ND_ was associated with steeper decrease in *R*_1_ in those same regions. All associations between baseline BP_ND_ and *R*_1_ longitudinal trajectories remained significant after correction for multiple testing. Figure 2 visualizes the association between baseline BP_ND_ and *R*_1_ over time.

**Table 3:** Associations with cross-sectional and longitudinal *R*_1_

	Region	Concurrent	Longitudinal
Model 1	Frontal	0.08 (0.07)	− 0.08 (0.03)*
	Temporal	− 0.07 (0.07)	− 0.12 (0.03)*
	Parietal	− 0.03 (0.07)	− 0.09 (0.03)*
	Occipital	0.14 (0.08)	− 0.12 (0.04)*
	Composite	− 0.07 (0.07)	− 0.11 (0.03)*
Model 2	Frontal	0.08 (0.07)	− 0.09 (0.03)*
	Temporal	0.01 (0.07)	− 0.12 (0.03)*
	Parietal	0.03 (0.07)	− 0.10 (0.03)*
	Occipital	**0.15 (0.08)**	− 0.14 (0.04)*
	Composite	− 0.003 (0.07)	− 0.11 (0.03)*

Results shown are beta (SE) as estimated by linear mixed models, outcome is *R*_1_. In model 1, the predictors are BP_ND_, time between PET scans in years, and the interaction BP_ND_ × time. Model 2 is additionally corrected for age, sex, and PET and MRI scanner type. Values given represent betas associated with BP_ND_ (concurrent) and BP_ND_ × time (longitudinal). Models included a random intercept. *R*_*1*_ and BP_ND_ are *z*-transformed

Bold *p* value < 0.05. *FDR corrected *p* value < 0.05

**Fig. 3:**
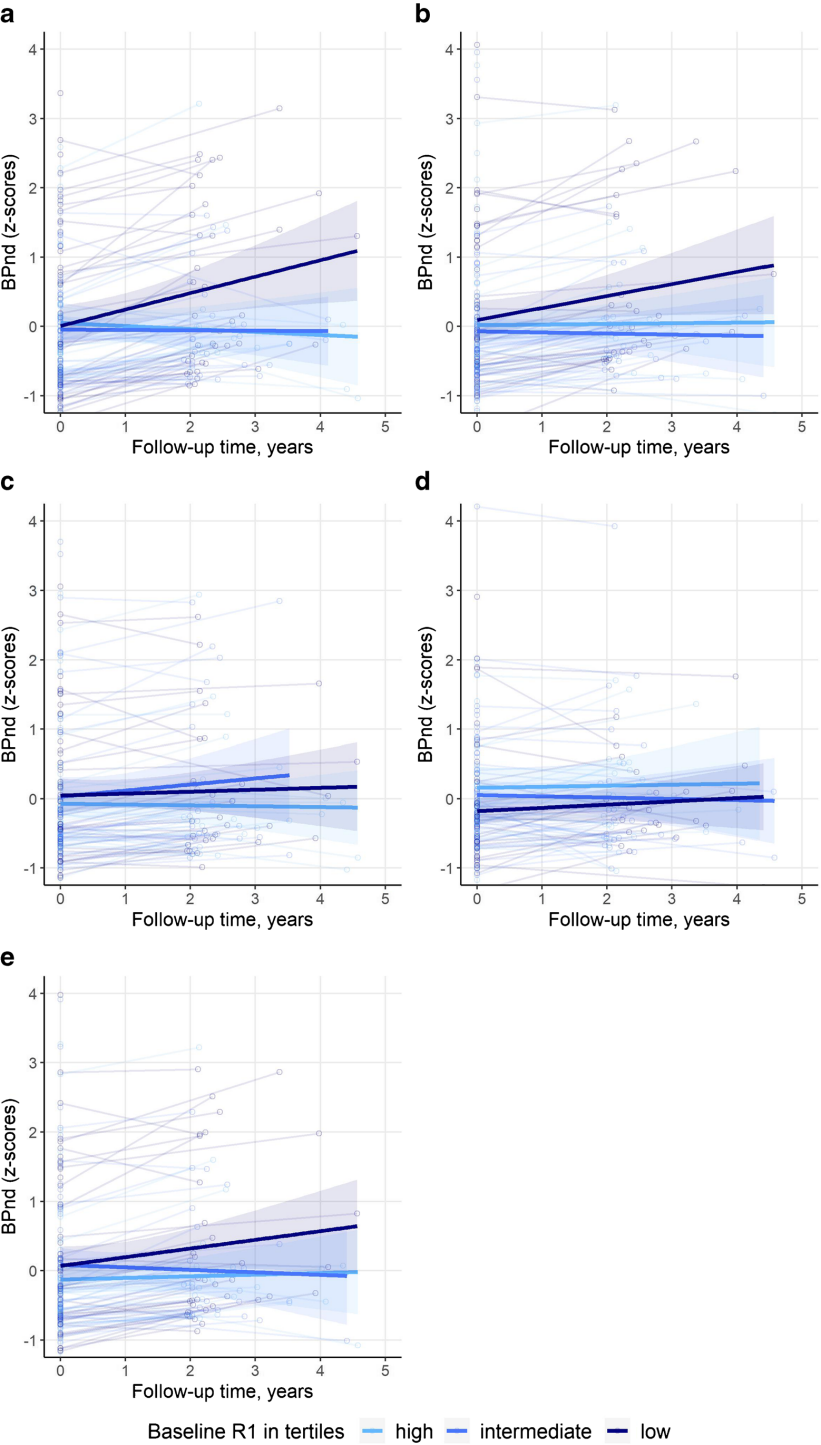
Visualization of trajectories of BP_ND_ over time. Spaghetti plots showing individual trajectories of BP_ND_ over time (**a** Frontal, **b** Temporal, **c** Parietal, **d** Occipital, **e** Composite regions). For visualization, the sample was divided into tertiles. Separate lines represent *R*_1_ tertiles (based on baseline values). BP_ND_ and *R*_1_ are z-transformed

Next, we investigated the effect of *R*_1_ on BP_ND_ (Table 4). Again, we found higher baseline values for occipital *R*_1_ to be associated with higher concurrent values for occipital BP_ND_, but no cross-sectional associations in other regions. Longitudinally, baseline *R*_1_ values in frontal, temporal, and composite regions were inversely associated with change in BP_ND_ over time in those same regions. This means a lower baseline *R*_1_ was associated with an increase in BP_ND_ over time. After correction for multiple testing, baseline *R*_1_ remained associated with longitudinal BP_ND_ in frontal and composite regions. Figure 3 visualizes the association between baseline *R*_1_ and BP_ND_ over time.

**Table 4:** Associations with cross-sectional and longitudinal BP_ND_

	Region	Concurrent	Longitudinal
Model 1	Frontal	0.09 (0.07)	− 0.08 (0.02)*
	Temporal	− 0.06 (0.07)	− **0.03 (0.01)**
	Parietal	− 0.01 (0.07)	− 0.02 (0.01)
	Occipital	**0.14 (0.07)**	0.0003 (0.02)
	Composite	− 0.06 (0.07)	− 0.04 (0.01)*
Model 2	Frontal	0.09 (0.08)	− 0.08 (0.02)*
	Temporal	0.04 (0.08)	− **0.03 (0.01)**
	Parietal	0.05 (0.08)	− 0.02 (0.01)
	Occipital	**0.18 (0.08)**	− 0.002 (0.02)
	Composite	0.02 (0.08)	− 0.04 (0.01)*

Results shown are beta (SE) as estimated by linear mixed models, outcome is BP_ND_. In model 1, the predictors are *R*_1_, time between PET scans in years, and the interaction *R*_1_ × time. Model 2 is additionally corrected for age, sex, and PET and MRI scanner type. Values given represent beta associated with *R*_1_ (concurrent) and *R*_1_ × time (longitudinal). Models included a random intercept. *R*_1_ and BP_ND_ are *z*-transformed

Bold *p* value < 0.05. *FDR corrected *p* value < 0.05

## Discussion

In this study in a relatively large sample of cognitively normal individuals, we found that low rCBF and high amyloid burden, both measured by dynamic [^18^F]florbetapir PET, were independently associated with worse trajectories for tests of memory and attention. Longitudinal imaging showed that despite the absence of cross-sectional associations, higher baseline amyloid burden was associated with a decline in rCBF over time, while at the same time, a low baseline rCBF was associated with increase in amyloid burden over time. This provides evidence that amyloid accumulation and reduced rCBF are parallel disease processes without fixed order in the cascade of events leading to cognitive decline.

We found that low baseline rCBF predicted worse performance over time on tests for memory and attention. After adding baseline amyloid burden as covariate, rCBF remained associated with cognitive slope. For one memory test, there seemed to be an interaction effect, as rCBF predicted cognitive slope mainly in individuals with low amyloid burden. Our results are in line with previous studies that showed low baseline CBF predicted future cognitive decline [23, 24], and clinical progression to MCI or dementia [38, 39]. We extend on these findings by showing rCBF adds unique predictive value in addition to amyloid burden, using an extensive neuropsychological test battery. Previous studies with a cross-sectional design found that lower baseline CBF was associated with worse concurrent cognitive performance in the entire disease spectrum [9, 11, 14, 40], but studies specifically investigating cognitively normal individuals report conflicting results [20, 22]. We did not find any cross-sectional associations between baseline rCBF and cognition. This is probably due to the fact that to be included in the SCIENCe project, our participants were extensively tested and judged to be cognitively normal, so variability in cognition at baseline is limited. This further highlights the importance of a longitudinal design with sufficient follow-up to study how brain changes contribute to cognitive decline in the earliest disease stages.

When we evaluated rCBF and amyloid burden longitudinally, the two measures were clearly associated, as high baseline amyloid burden was associated with a decline in rCBF, while vice versa and contrary to our hypotheses, a low baseline rCBF was also associated with an increase in amyloid burden. There are a number of possible explanations for these observations. According to the amyloid cascade hypothesis, and as translated to living humans in the hypothetical biomarker model, amyloid accumulation is among the first changes related to AD. This subsequently leads to tau deposition and then to neuronal injury. When we interpret rCBF as a measure of neuronal injury, we would expect to observe reduced rCBF downstream of amyloid. Our observation that high baseline amyloid burden predisposes for decline over time in rCBF is in line with this hypothesis [1]. They are also in line with studies suggesting low CBF to reflect decreases in metabolism and synaptic failure, because this places a decreasing CBF towards the end of the pathophysiology of AD [7, 8]. However, we also found low rCBF values in individuals with low amyloid burden, associated with subsequent increase in amyloid burden. This does not fit within this hypothetical sequence. Alternative hypotheses on the origin of AD include the two-hit vascular hypothesis, which poses that damage to the microcirculation of the brain resulting from vascular risk factors (hit one) would lead to reduced CBF [6, 41]. The compromised vessels would than lead to suboptimal breakdown and clearance of amyloid, which would result in amyloid accumulation (hit two). In this scenario, amyloid accumulation would be a downstream effect of reduced CBF. Our second observation, that low baseline rCBF also predisposes for an increase in amyloid burden over time, is in line with this second hypothesis. The probabilistic model of AD recognizes three variants of the disease and highlights that AD is a very complex disease and many factors are related to cognitive decline [42]. To add to this complexity, neurodegenerative pathologies other than AD are also related to cognitive decline. In our sample, one individual showed clinical progression to dementia with Lewy bodies and another to vascular dementia. Furthermore, mixed pathologies in neurodegenerative disease are not uncommon. For example, AD pathology is seen frequently in combination with alpha-synuclein inclusions, or vascular pathology [43]. Therefore, pathologies other than AD could also have contributed to our results. Overall, our results provide evidence for different sequences of events, and show that different factors contribute to cognitive decline, which underlines the complexity of cognitive decline.

In contrast to our longitudinal findings, we did not find cross-sectional associations between rCBF and amyloid burden, except in the occipital region, in which a higher amyloid burden was subtly associated with a higher rCBF. This might be interpreted as overcompensation, yet we feel that this result should not be overinterpreted, since the association did not survive correction for multiple testing. Previous studies in cognitively normal individuals found contradictory results. Some found lower CBF in amyloid positive individuals [18], others a higher CBF [15], or no amyloid-related differences in CBF at all [20, 21]. The lack of cross-sectional associations in our sample of cognitively normal individuals, in combination with the inconsistencies in literature, highlights the importance of longitudinal data to study how different measures of brain pathology contribute to cognitive decline and dementia. Pathology accumulates in the course of many years, and this further stresses the relevance of well-phenotyped clinical cohorts with sufficiently long duration of follow-up.

Our study is among the first to investigate the longitudinal relationships between amyloid burden and rCBF. Only one other study in 28 nondemented elderly investigated change in CBF using [^15^O]H_2_O PET in relation to amyloid status, and found both increases and decreases in CBF in amyloid-positive individuals compared to amyloid-negative individuals [16], This study concluded that the decrease in CBF in amyloid-positive individuals represented a response to neuronal insult as a result of amyloid deposition, providing evidence for the theory that amyloid accumulation predisposes for a reduction in CBF. They interpreted the increases as a representation of a compensational effect by attempting to preserve neuronal function. The latter could be viewed in line with our cross-sectional finding of a high amyloid burden related to a high rCBF in the occipital region. However, we feel that this result should not be overinterpreted. Furthermore, there are a number of differences with our study. First, this study investigated CBF but not amyloid deposition longitudinally. Also, this study acquired images for 60 s once the total radioactivity counts in the brain reached threshold levels. This is a semi-quantitative method for analysis of [^15^O]H_2_O PET data, which may have compromised the CBF measurements. Last, we used a much larger sample size, which makes our results more robust.

Our study has important implications. We show that amyloid accumulation and reduction in rCBF are separate and parallel disease processes which independently contribute to cognitive decline while influencing each other longitudinally, without ordering one process before the other. This provides additional insight in the pathophysiology of AD. Our results suggest that in the hypothetical biomarker model of amyloid accumulation leading to tau pathology, neuronal injury, and cognitive decline, changes in CBF could be placed before or after amyloid accumulation. Since other studies also show tau pathology and rCBF are independently associated with cognition in AD, rCBF probably reflects a different aspect of AD pathology [44]. It furthermore illustrates there are multiple pathways through which AD biomarkers can become abnormal.

Strengths of our study include that we had a relatively large sample of cognitively normal individuals with considerable follow-up. We show that by using one dynamic [^18^F]florbetapir PET scan, information about two relevant biomarkers can be obtained, which both provide predictive information about future cognitive decline. Additionally, we had repeated PET scans available for a large group of participants, enabling the investigation of longitudinal trajectories of rCBF and amyloid burden. Limitations of our study include that we did not take all variables into account that potentially affect rCBF, such as diurnal variations, genetic factors, and cerebrovascular risk factors. Last, although we already had a relatively long follow-up duration of 3.8 years on average, an even longer period would enable us to capture more clinically relevant changes in cognition, in this sample of cognitively normal individuals.

Concluding, we showed that amyloid burden and rCBF were independently associated with cognitive decline over time in a group of initially cognitively normal individuals. Even though there were hardly any cross-sectional relationships between amyloid burden and rCBF, we observed an association between high baseline amyloid burden and a subsequent decrease in rCBF, and inversely an association between a low baseline rCBF and a subsequent increase in amyloid burden. Our results provide evidence that amyloid accumulation and decrease of CBF are separate and parallel disease processes, each of them providing unique predictive information, and enhancing the other longitudinally.

## Data Availability

The data that support the findings of this study may be shared upon reasonable request.
